# Sex-Specific Associations Between 2D:4D Digit Ratio and Physical Fitness in Prepubertal Children: Evidence from Standardized Agility, Strength, and Endurance Assessments

**DOI:** 10.3390/children12101391

**Published:** 2025-10-15

**Authors:** Fatih Akgül, Ahmet Kurtoğlu, Rukiye Çiftçi, Özgür Eken, Bekir Çar, Alperen Şanal, Monira I. Aldhahi

**Affiliations:** 1 Department of Physical Education and Sport Teaching, Faculty of Sports Sciences, Yozgat Bozok University, Yozgat 66100, Turkey; fatih.akgul@bozok.edu.tr; 2 Department of Coaching, Faculty of Sport Science, Bandirma Onyedi Eylul University, Balikesir 10200, Turkey; akurtoglu@bandirma.edu.tr; 3 Department of Anatomy, Medical Faculty, Gaziantep Islamic Science and Technology University, Gaziantep 28010, Turkey; rukiye.ciftci@gibtu.edu.tr; 4 Department of Physical Education and Sport Teaching, Faculty of Sports Sciences, Inonu University, Malatya 44280, Turkey; ozgur.eken@inonu.edu.tr; 5 Department of Physical Education and Sport Teaching, Faculty of Sport Sciences, Bandirma Onyedi Eylul University, Balikesir 10200, Turkey; bcar@bandirma.edu.tr; 6 Department of Sport Sciences, Institute of Graduate Studies, Çanakkale Onsekiz Mart University, Çanakkale 17020, Turkey; alperensanal48@gmail.com; 7 Department of Rehabilitation Sciences, College of Health and Rehabilitation Sciences, Princess Nourah bint Abdulrahman University, P.O. Box 84428, Riyadh 11671, Saudi Arabia

**Keywords:** 2D:4D digit ratio, prenatal androgen exposure, prepubertal fitness, physical performance, agility, muscular endurance, standing long jump, sex differences

## Abstract

**Highlights:**

**What are the main findings?**

- In boys, higher 2D:4D ratios were modestly linked to better bent-arm hang performance and faster agility times.
- No significant associations were found in girls, highlighting the subtle sex-specific influences of prenatal hormones on childhood fitness.

**What is the implication of the main finding?**

- Prenatal hormonal environment may contribute subtly to physical performance traits in early childhood.
- These associations are not strong enough for talent identification but may guide future longitudinal and developmental research.

**Abstract:**

**Background:** The second-to-fourth digit ratio (2D:4D) serves as a non-invasive proxy for prenatal androgen exposure. While its relationship with adult athletic ability is well documented, evidence for its association with childhood physical fitness remains inconsistent, and links between 2D:4D and objective fitness measures in prepubertal children are unclear. **Methods:** In this cross-sectional study, 338 prepubertal children (181 girls, 157 boys; aged 5–12 years) underwent precise measurement of right- and left-hand 2D:4D ratios (intra-class correlation coefficient = 0.94). Physical fitness was evaluated using standardized tests: the Illinois agility run, bent-arm hang, and standing long jump. **Results:** Among boys, higher 2D:4D ratios were modestly associated with prolonged bent-arm hang performance (β = 0.19, q = 0.04) and shorter Illinois agility times (β = −0.19, q = 0.04). No significant associations were observed in girls. All effect sizes were small, suggesting subtle, sex-dependent influences rather than robust predictors of performance. **Conclusions:** These findings indicate that prenatal hormonal environment may exert a limited, sex-specific influence on early physical fitness characteristics. Although biologically informative, the observed associations are insufficient for direct application in talent identification in sports. Longitudinal research incorporating direct hormonal measurements and broader populations is recommended to clarify developmental mechanisms and causal pathways.

## 1. Introduction

Prenatal androgen exposure has been proposed to influence musculoskeletal development, neuromotor coordination, and later physical performance capacities. The second-to-fourth digit ratio (2D:4D) is a widely accepted, non-invasive proxy for this prenatal hormonal milieu: lower ratios are generally indicative of higher prenatal testosterone, whereas higher ratios suggest greater prenatal estrogen exposure. Because the 2D:4D ratio stabilizes early in life and remains relatively constant throughout adolescence and adulthood, it serves as a reliable marker of developmental biology and sexual dimorphism [1,2]. Prenatal sex steroids have a significant impact on finger development, demonstrating a negative correlation between 2D:4D and fetal testosterone/fetal estrogen. Three conditions can be considered when formulating the length of the index finger in the human hand: the index finger is shorter than the ring finger (2D < 4D), the index finger is equal to the length of the ring finger (2D = 4D), and the index finger is longer than the ring finger (2D > 4D). In this formulation, (2D < 4D) is generally dominant in boys, and (2D > 4D) is generally dominant in girls, although 2D = 4D also occurs. In other words, the gene for a short index finger is dominant in boys and recessive in girls. The presumed relationship between 2D:4D and prenatal testosterone led Manning and Bundred to suggest that high athletic performance may be associated with a low 2D:4D [3]. 2D:4D is a biomarker for performance in various sports, athletic disciplines (e.g., middle- and long-distance running), and cardiovascular disease [4].

Association between 2D:4D and athletic performance has been documented repeatedly. Meta-analyses and large-scale studies report significant but small correlations between digit ratios and traits such as muscular strength, agility, and endurance across adolescent and adult populations [5,6,7,8,9]. However, these effects may be age-dependent, as prepubertal children differ from adults in terms of maturational status, neuromuscular coordination, and endocrine dynamics, potentially modifying the expression of 2D:4D–performance relationships. While it does not determine the performance outcomes of 2D:4D, it offers a significant contribution as a potential indicator. It is also important to note that children’s physical development is significantly influenced by environmental, socioeconomic, and educational factors.

Childhood physical fitness is a robust predictor of long-term health, motor skill development, and future sport specialization [10]. At the same time, global cardiorespiratory fitness among children and adolescents has declined by approximately 0.43% annually over recent decades [11]. Understanding whether prenatal hormonal factors contribute—albeit modestly—to early fitness phenotypes may help disentangle immutable biological influences from modifiable environmental determinants.

There are studies in the literature examining the relationship between the 2D:4D ratio and physical performance in children. Crewther et al. demonstrated that 2D:4D combined with testosterone predicted vertical jump performance in athletic boys [12], whereas Nobari et al. found no meaningful associations between 2D:4D and sprinting, change-of-direction ability, or aerobic fitness in elite youth soccer players [13]. Similarly, Azam et al. observed only weak relationships between digit ratio and anaerobic-based fitness in school-aged children [14]. These discrepancies suggest that sex-specific effects, sample heterogeneity, and methodological differences may account for divergent findings and highlight the need for large samples, objective tests, and rigorous statistical control.

Proposed mechanisms include androgen receptor sensitivity, musculoskeletal differentiation, and neuromotor coordination pathways, which may generate subtle, sex-dependent performance correlates in childhood [9,15,16].

The inconsistencies across previous studies may stem from several methodological limitations. One key factor is the measurement technique used to assess 2D:4D, which varies between direct caliper-based methods and indirect approaches using photocopies or scans. Direct measurements are generally more precise and less prone to distortion caused by finger pressure or image scaling, potentially explaining variation in reported associations. Another source of discrepancy is sample composition: studies involving elite youth athletes [13] differ markedly from those assessing general or mixed populations [12,14], leading to differences in maturational status, training exposure, and hormonal profiles. Finally, small sample sizes in some investigations may have limited statistical power to detect small but meaningful correlations. Addressing these methodological inconsistencies, the present study employed a standardized direct measurement protocol and tested a sufficiently powered cohort of prepubertal children to clarify sex-specific relationships between 2D:4D and objective physical fitness parameters.

Accordingly, this study investigated sex-specific associations between 2D:4D and physical fitness in a large sample of prepubertal children using standardized assessments of agility, muscular endurance, and explosive strength. We therefore hypothesized that 2D:4D would exhibit small, sex-specific associations with objective fitness measures in prepubertal children, with detectable effects more likely in boys than in girls.

## 2. Materials and Methods

### 2.1. Research Design & Participants

This study employed a cross-sectional observational design to investigate sex-specific associations between the second-to-fourth digit ratio (2D:4D) and objective physical fitness measures in prepubertal children. A total of 338 participants (181 girls, 157 boys) aged 5–12 years were recruited from local primary schools and sports academies. Eligibility criteria included: (i) absence of any musculoskeletal, cardiovascular, or metabolic condition that could impair testing or increase risk; and (ii) the ability to complete all assessments within a single session. Children were excluded if they declined participation, withdrew assent, or presented with injuries or chronic illnesses preventing safe testing.

The sample size exceeded the a priori power requirement determined by G*Power (v3.1.7) for multiple linear regression (f^2^ = 0.035, α = 0.05, 1 − β = 0.80), ensuring sufficient power to detect small effect sizes after covariate adjustment. Participant sex and age distributions were balanced to allow meaningful sex-stratified analyses.

The study protocol received ethical approval from the Bandırma Onyedi Eylül University Health Sciences Ethics Committee (approval no. 2023/24) and was conducted in accordance with the Declaration of Helsinki. Written informed consent was obtained from parents or legal guardians, and verbal assent was obtained from all children prior to participation. Data collection was performed in a controlled indoor environment to minimize environmental variability.

### 2.2. Procedure

All evaluations were conducted in a closed area with a standard hard court surface, at a temperature of 20–24 °C and relative humidity of 50–55%, to minimize the effect of external factors. Ambient temperature and humidity were measured using a thermohygrometer (Testo 608-H2, Testo SE & Co. KGaA, Titisee-Neustadt/Lenzkirch, Germany). Tests were not conducted if these ratios were lower or higher than specified. Testing for each participant was completed within a single 90 min session. Upon arrival, participants performed a 10 min standardized warm-up (3 min light jogging, 4 min dynamic mobility drills, 3 min test-specific practice movements). Following the warm-up protocol, participants underwent a 20 m sprint, sit-up test, sit and reach test, push-up test, standing long jump (SLJ) test, vertical jump test, and hanging with bent-arm (HBA) test.

To minimize the effects of fatigue, the tests were completed over two days. On the first day, following the same warm-up protocol, the 20 m sprint, sit-up test, and push-up test were performed. On the second day, the SLJ, sit-reach test, vertical jump, and HBA tests were performed in that order. A minimum of 5 min’ rest was provided between each test. After each analysis, the perceived exertion level (RPE) was assessed subjectively. Those with an RPE < 3 were allowed to proceed to the next measurement. All instructions, demonstrations and monitoring were provided by trained assessors using standard verbal cues to ensure consistent test conditions for all participants. All tests were conducted at the same time of day for each participant (10:00–12:00 before noon). Participants were asked to refrain from consuming food and drink other than water for at least 3 h prior to the test. Before the tests, participants and their parents were warned to avoid foods that could negatively affect performance, such as coffee and fatty foods.

### 2.3. Data Collections

Rating of Perceived Exertion (RPE): The Borg RPE scale was used to determine participants’ fatigue levels between trials. Participants rated the scale, which ranged from 0 = ‘not at all difficult’ to 10 = ‘extremely difficult’. All participants attended a practice session prior to the test day. RPE was verbally administered to participants at the end of the rest period following each test and recorded. An RPE < 3 threshold was set at the end of the rest period for transition to the next trial. Participants with RPE > 3 were given an additional 2–3 min rest period.

Twenty-Meter Sprint Test (20 m): Prior to the speed tests, two pairs of photocells (Smart Speed, Fusion Equipment, AUS) were placed at distances of 0 and 20 m on the running track. Participants started on their own from a half-crouching position 0.3 m behind the starting line and performed two sprints. After the practice phase, each participant was given two attempts. Participants were tested in groups of 10, and after each participant completed the first test, the first participant was taken back for the second test. A rest interval of at least 5 min was provided between the two tests, and the best trial was recorded [17].

Flexibility Test: Participants were seated on the floor with bare feet flat against a testing bench. They were instructed to stretch their torso forward as far as possible while keeping their arms and fingers extended and straight and holding the furthest position for one–two seconds. After two attempts, the best results were recorded for analysis [18].

Push-up Test: Participants assumed a full push-up position with straight arms and rigid bodies. They were instructed to lower themselves until their chest nearly touched the ground and then return to the starting position with fully extended arms. Participants performed as many push-ups as possible within 30 s, and the total number completed was recorded for analysis [19].

Sit-Up Test: Participants lay supine on a gymnastics mat with their hands clasped behind their necks and feet flat on the mat, with their knees bent at a 90° angle. The helper ensured that their feet remained in contact with the mat. Participants were instructed to perform as many sit-ups as possible within a 30 s period, and the total number of sit-ups completed was recorded [20].

Vertical Jump Test: The vertical jump performance of the athletes was measured using an electronic Smart Speed Lite (VALD Performance, Queensland, Australia) system. Participants were instructed to jump as high as possible when ready and then land back on the mat. Jump heights were measured electronically in centimeters, and the best result out of the three attempts was recorded for analysis [21].

Standing Long Jump Test: This test required the participants to place their toes behind a designated starting line with a measuring tape aligned between their feet. They were instructed to jump as far as possible from the standing position. The distance was measured from the starting line to the point where the back of the heels landed. To ensure reliability, each participant completed the test twice and the best distance was recorded for analysis [22].

Bent-Arm Hang Test: Participants’ hang time on the pull-up bar was measured under the supervision of the test leader to prevent any swinging motion. The participants gripped the bar with shoulder width and overhand grip. The timer was started as soon as the participant’s chin passed the bar, and the timer was stopped when the participant could no longer maintain the position and their eyes dropped below the bar level. The duration was recorded in seconds [23].

Measurement of Physical Parameters and Test Conditions

The validity of the test results in this study depended heavily on the objective and trustworthy measurement of the participants’ physical characteristics. To ensure the validity and reliability of the test results, participants’ anthropometric characteristics (height and body mass) were measured once before the testing sessions under standardized conditions. Each physical fitness test was performed twice (for the 20 m sprint, standing long jump, and sit-and-reach test) or three times (for the vertical jump test), and the best score was recorded for analysis. Adequate rest intervals (at least 3 min) were provided between attempts to minimize fatigue effects:

Height: A conventional stadiometer was used to measure each participant’s height to an accuracy of 0.1 cm. Accuracy and repeatability of measurements are guaranteed by this technique.

Body Mass (Weight): Using a calibrated digital scale, body mass was calculated with an accuracy of 0.1 kg.

2D:4D Finger Ratio: Using a precision caliper or ruler, the lengths of each participant’s index finger (second finger) and ring finger (fourth finger) were measured to an accuracy of 0.1 mm. The measurements were performed by two researchers using the same caliper, and two measurements were taken for each finger. The average of the measurements taken by both researchers was calculated and the results were recorded. The purpose of recording these measurements was to investigate behavioral and biological relationships.

Evaluation of Test Conditions and Physical Parameters

The validity of the test results in this study depended heavily on the objective and trustworthy measurement of the participants’ physical characteristics.

Environmental Factors and Test Duration

To maintain methodological consistency, all tests were run in a single session. The duration of each exam session was roughly ninety minutes. In order to reduce participants’ physical and mental exhaustion and avoid a drop in test performance, standard rest periods were offered in between examinations. To reduce the possible influence of outside environmental influences on the test findings, the temperature and humidity levels of the enclosed space where the tests were carried out were regulated. The dependability of the measured parameters and test findings was improved under these controlled settings.

### 2.4. Statistical Analysis

In this study, statistical analyses were performed using SPSS 25 software. First, the normality of the data was tested using the Kolmogorov–Smirnov test. Here, Skewness Kurtosis values were examined for values with *p* < 0.05 (−1.5 and +1.5). Levene’s test results were examined for homogeneity of variance. In this study, the demographic characteristics of the participants according to sex and baseline performance parameters were tested using an Independent Sample T-test. The relationship between the 2D:4D ratio of male and female participants and 20m sprint, SR, push-up, sit-up, SLJ, VJ, Illinois, and HBA tests was tested using Pearson Correlation Analysis. To control the false discovery rate (FDR) in multiple comparisons, the Benjamini–Hochberg correction was applied, and the significance level was set at a corrected *p* (q) < 0.05. JASP software (version 0.19.3) (University of Amsterdam, Amsterdam, The Netherlands) was used to visualize the correlation analysis. Cohen’s d test was used to determine the effect size in this study. According to this test, effect size reference intervals were taken as <0.1 = trivial, 0.2 to 0.5 = medium effect, 0.6 to 1.0 = large effect [24]. The significance level was set as 0.05.

## 3. Results

Table 1 presents the demographic and baseline characteristics of the participants. Accordingly, there was no significant difference in age, height, body weight, BMI, 2D, 4D, 2D:4D ratio, SLJ, VJ, and HBA between the sexes (*p* > 0.05). However, there were no significant differences between genders in 20m sprint performance [t = −3.533, Cohen’s d = −0.47 (medium effect), *p* < 0.001], SR [t = −6.076, Cohen’s d = −0.76 (high effect), *p* < 0.001], push-up [t = 2.172, Cohen’s d = 0. 33 (medium effect), *p* = 0.031], sit-up [t = 2.831, Cohen’s d = 0.36 (medium effect), *p* = 0.005], and Illinois test [t = −5.467, Cohen’s d = −1.11 (large effect), *p* < 0.001].

**Figure 1:**
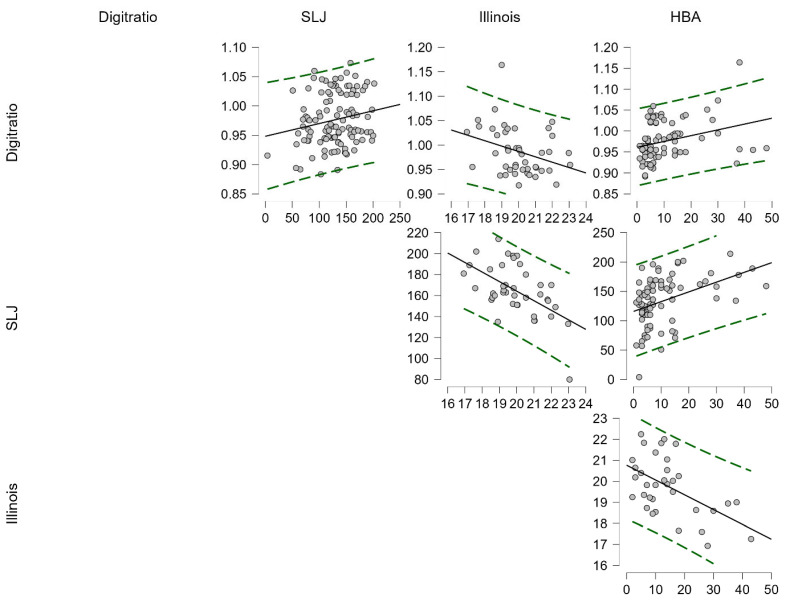
Pearson Correlation Analysis Results between 2D:4D ratio and SLJ, Illinois, and HBA of Boy Participants.

In Table 2, the relationship between the digit (2D:4D) ratio and the performance parameters of male participants is analyzed. Accordingly, there was a positive correlation between the 2D:4D ratio and SLJ (r = 0.191, *p* = 0.044) and HBA (r = 0.304, *p* = 0.007) and a negative correlation with Illinois (r = −0.326, *p* = 0.029) (Figure 1).

The relationships between the 2D:4D digit ratio and various physical performance parameters were examined in the girls (Table 3). No significant correlation was found between the 2D:4D ratio and any performance measures. Specifically, the 2D:4D ratio was not significantly correlated with the 20 m sprint, sit and reach, push-up, sit-up, standing long jump, vertical jump, Illinois agility test, or hanging with bent arms. These findings contrast with those in males, highlighting potential sex differences in the relationship between early androgen exposure and the development of physical performance during childhood.

## 4. Discussion

This study investigated the relationship between the second-to-fourth digit ratio (2D:4D) and various physical performance metrics in children aged 5–12 years. Our findings provide important insights into the potential link between prenatal hormonal influence and physical fitness during childhood. Specifically, we observed sex-specific correlations between the 2D:4D ratio and certain performance parameters, most notably in the male participants.

For male participants, we found significant positive correlations between the 2D:4D ratio and standing long jump (SLJ) and hanging with bent arms (HBA), while a negative correlation was observed in the Illinois agility test. A higher 2D:4D ratio in male children is known to be generally linked to low levels of testosterone during pregnancy; however, this ratio may also be linked to improved performance in tasks that require strength and endurance, such as the hanging test with bent arms. Conversely, an inverse relationship has been noted in tests that require agility, such as the Illinois agility test. In this context, a high 2D:4D ratio would be expected to contribute to increased agility performance in males, given the Illinois test is one where activities accomplished in a shorter amount of time indicate better performance. However, the results revealed that boys with higher 2D:4D ratios tended to have slower agility times on the Illinois test, indicating lower agility performance. This negative association contrasts with some previous findings, which generally report better performance with lower 2D:4D ratios. Such paradoxical outcomes suggest that the relationship between 2D:4D and physical fitness may depend on task type, developmental stage, and sample characteristics, indicating a nuanced and context-dependent influence of prenatal hormonal exposure on agility performance [8,9,12,13,25]. Interestingly, the negative association between the 2D:4D ratio and agility performance in boys appears paradoxical given that lower 2D:4D ratios, typically linked to higher prenatal testosterone, are often associated with enhanced motor ability [1,9,13]. Several mechanisms may explain this discrepancy. One possibility is that neuromotor maturation and coordination efficiency develop asynchronously during prepubertal years, such that boys with relatively higher 2D:4D ratios—possibly reflecting slower maturational tempo—may temporarily display differences in movement control that affect agility tasks requiring rapid directional changes [26]. Another explanation could involve task specificity and biomechanical factors: the Illinois agility test integrates balance, reaction time, and directional precision, components that depend more on neuromotor coordination than on maximal strength or power [27,28]. Thus, the link between 2D:4D and agility may not mirror relationships observed in purely power-based or endurance-based tasks [5,13]. Finally, measurement variability and individual differences in limb proportions, growth stage, or motivation could modestly influence test outcomes, contributing to apparent paradoxes in correlational data [1,8]. Collectively, these findings underscore that 2D:4D–performance relationships in childhood likely reflect a complex interplay of biological maturation, hormonal background, and task-specific neuromotor demands. The current findings may also be interpreted within the broader endocrine framework proposed by Jürimäe et al. [29], who demonstrated associations between 2D:4D ratios and circulating levels of ghrelin, leptin, and IGF axis hormones in young male and female swimmers. These results indicate that 2D:4D may not only reflect prenatal androgen exposure but could also be linked to ongoing hormonal regulation of growth and metabolism during childhood and adolescence. This aligns with our observation that 2D:4D showed modest, sex-specific relationships with strength and agility, suggesting a multifactorial hormonal basis for physical performance in prepubertal children [29]. In our study, we observed a significant relationship between the 2D:4D ratio and certain physical performance parameters in boys, while Crewther et al. (2022) found a strong link between the 2D:4D ratio, testosterone, and jump performance in athletic boys [12]. Both studies emphasized the role of prenatal androgen exposure in physical fitness, particularly in tasks involving strength and endurance. However, while our study focused on general childhood populations, Crewther et al. highlighted the importance of testosterone’s activational and organizational effects in athletic boys, adding complexity by modeling the nonlinear nature of testosterone’s impact on jump performance. Kobus et al. (2021) found that the 2D: 4D ratio is associated with birth weight and muscle strength, with a negative correlation specifically noted for birth weight [25]. Similarly, our study identified positive correlations between the 2D:4D ratio and physical performance parameters, particularly the standing long jump and bent-arm hang tests, among boys. However, a significant difference between the two studies was that we found no significant association in girls, whereas Kobus et al. identified significant correlations between the 2D:4D ratio and muscle strength in both sexes. These discrepancies suggest that the effect of prenatal sex hormones on postnatal physical performance may vary depending on age and sex. Nobari et al. (2023) explores this relationship in the context of elite U-14 soccer players, finding no significant correlations between 2D:4D and key fitness metrics such as VO2max, change of direction (COD), or sprint ability [13]. This highlights potential limitations due to the small sample size and maturational heterogeneity among the participants, suggesting that the influence of 2D:4D on physical performance may not manifest until later developmental stages. Conversely, our study focused on a broader age range of children (5–12 years) and observed significant sex-specific correlations, particularly among boys. Our study found positive associations between 2D:4D and tasks requiring strength and endurance, such as standing long jump and hanging with bent arms, while noting a lack of such correlations in female participants. These contradictory results demonstrate the intricate relationships among age, gender, and prenatal hormone exposure that affect athletic performance. They also imply that the impact of the 2D:4D ratio may differ based on developmental stages as well as the particular physical examinations conducted. Pasanen and colleagues’ systematic review and meta-analysis, which focused on handgrip strength as one indicator of muscle strength, revealed a weak negative correlation between muscle strength and the 2D:4D ratio. This suggests that higher muscle strength capacity is linked to lower 2D:4D ratios [8]. This study highlights the impact of prenatal testosterone exposure on physical abilities, first by focusing on a broader range of fitness measures in a younger population, and second by reinforcing findings on strength in a more diverse age group. These findings suggest that the 2D:4D may be a more consistent predictor of muscle strength in certain contexts but may also play a more nuanced role in tasks requiring coordination and endurance, particularly in children. Sorokowski and Kowal (2024) conducted a meta-analysis examining the link between the 2D:4D ratio and testosterone levels, and its potential as a predictor of various hormonal changes, particularly during adulthood [9]. In contrast, our study focused on children, specifically analyzing sex-specific correlations between 2D:4D and physical performance parameters such as sprint, sit-ups, and agility. While both studies highlighted the role of prenatal hormonal exposure, a child study observed sex-specific differences in boys, suggesting that 2D:4D could predict performance in tasks involving strength and endurance. In contrast, the meta-analysis found inconsistent evidence for the relationship between the digit ratio and testosterone, emphasizing the need for caution in using 2D:4D as a proxy for hormonal exposure in broader populations. These findings indicate that developmental stage, age, and sex may influence the manifestation of 2D:4D-related effects on performance. This finding aligns with prior research suggesting that the 2D:4D ratio may serve as an indicator of prenatal hormonal influence on physical abilities, particularly in tasks related to muscular strength and coordination [6,14].

In contrast, we did not observe significant correlations between the 2D:4D ratio and performance metrics in the female participants. This lack of association suggests that the relationship between 2D:4D and physical performance is more pronounced in boys, potentially due to the greater variability in prenatal androgen exposure among males. It is also possible that other factors, such as environmental influences or developmental differences, play a more substantial role in determining the physical performance of girls.

The absence of significant associations in girls and the presence of correlations in boys raises intriguing questions regarding the role of sex hormones in shaping physical performance across different developmental stages. Previous studies on adults have predominantly focused on male participants and our findings suggest that similar sex-specific patterns may emerge during childhood. However, further research is needed to fully understand the mechanisms underlying these differences.

Another important finding is the positive correlation between 2D:4D and hanging with bent arms (HBA), a test requiring upper-body strength and endurance, in boys. This result contrasts with those of some studies conducted in adult populations, where a lower 2D:4D ratio (indicative of higher prenatal testosterone exposure) was often associated with superior physical performance [15,16]. This discrepancy may be due to developmental changes in hormonal influences or the nature of the tasks being measured. Childhood fitness assessments, such as hanging with bent arms, may rely more on coordination and endurance than raw strength measures typically examined in adults.

It is also important to consider the magnitude of the observed correlations. Although several associations between 2D:4D and physical fitness indices reached statistical significance, the corresponding r-values were small (ranging from approximately 0.15 to 0.28). These values indicate weak-to-moderate effects, consistent with prior evidence suggesting that 2D:4D–performance relationships typically fall within this range (r ≈ 0.1–0.3; [1,9,24]). Therefore, while the current findings may reflect biologically meaningful trends, their practical significance should be interpreted cautiously. The weak effect sizes likely reflect the multifactorial nature of motor performance in children, which depends not only on prenatal hormonal factors but also on neuromotor development, environmental stimulation, and training exposure.

Another explanation could involve task specificity and biomechanical factors: the Illinois agility test integrates balance, reaction time, and directional precision, components that depend more on neuromotor coordination than on maximal strength or power [30,31]. Thus, the link between 2D:4D and agility may not mirror relationships observed in purely power-based or endurance-based tasks [32,33]. Finally, measurement variability and individual differences in limb proportions, growth stage, or motivation could modestly influence test outcomes, contributing to apparent paradoxes in correlational data [34,35]. Collectively, these findings underscore that 2D:4D–performance relationships in childhood likely reflect a complex interplay of biological maturation, hormonal background, and task-specific neuromotor demands.

Our study had some limitations that should be considered when interpreting the results. This cross-sectional design limits our ability to draw causal conclusions regarding the relationship between prenatal hormone exposure and childhood fitness. Longitudinal studies tracking changes in 2D:4D ratios and performance over time could provide a clearer understanding of how these associations evolve. Additionally, although we included a large sample of children, further research involving diverse populations and more refined measures of hormonal exposure may enhance the generalizability of our findings.

## 5. Conclusions

This cross-sectional study identified small but statistically significant sex-specific associations between the 2D:4D ratio and selected physical fitness measures in prepubertal children. In boys, a higher 2D:4D ratio was modestly associated with improved bent-arm hang performance and faster agility times, whereas no meaningful relationships were observed in girls. These findings support the notion that prenatal hormonal exposure may subtly influence early neuromuscular and motor performance capacities, although the observed effects were limited in magnitude.

The moderate sample size and cross-sectional nature of this study restrict causal inference. Therefore, while the 2D:4D ratio may provide insight into developmental and endocrine mechanisms underlying motor function, it should not be interpreted as a determinant of athletic potential. Future longitudinal and mechanistic studies incorporating direct hormonal markers, maturational status, and environmental factors are needed to clarify how early biological influences interact with training and growth to shape long-term physical performance trajectories. 

## Figures and Tables

**Table 1:** Demographic and baseline characteristics of participants.

Parameters	Boys *n* = 157	Girls *n* = 181	*p*-Value	ES	%95 CI	
					Lower	Upper
Age (year)	6.52 ± 4.40	6.38 ± 4.17	0.766	0.033 (small)	−0.78	1.06
Weight (kg)	31.40 ± 13.75	33.71 ± 40.03	0.505	−0.075 (small)	−9.10	4.48
Height (cm)	131.35 ± 18.90	129.37 ± 15.51	0.309	0.116 (small)	−1.78	5.75
BMI (kg/m^2^)	17.32 ± 2.92	17.13 ± 2.97	0.576	0.06 (small)	−0.46	0.83
2D (cm)	5.36 ± 0.81	5.30 ± 0.74	0.512	0.074 (small)	−0.11	0.23
4D (cm)	5.50 ± 0.83	5.40 ± 0.77	0.303	0.115 (small)	−0.08	0.26
2D:4D	0.97 ± 0.05	0.98 ± 0.05	0.272	−0.123 (small)	−0.01	0.005
20 m Sprint (s)	4.40 ± 0.73	4.81 ± 0.96	<0.001	−0.471 (medium)	−0.63	−0.18
SR (cm)	18.55 ± 6.44	23.45 ± 6.41	<0.001	−0.762 (large)	−6.49	−3.31
Push-up (rep)	7.68 ± 7.92	5.35 ± 5.90	0.031	0.334 (medium)	0.21	4.44
Sit-up (rep)	27.04 ± 27.05	19.33 ± 14.30	0.009	0.368 (medium)	2.34	13.07
SLJ (cm)	129.30 ± 39.05	121.84 ± 30.76	0.095	0.214 (medium)	−1.14	16.06
VJ (cm)	21.62 ± 10.14	20.46 ± 7.11	0.283	0.135 (small)	−0.96	3.29
Illinois (s)	19.98 ± 1.42	22.10 ± 7.11	<0.001	−1.116 (large)	−2.89	−1.35
HBA (s)	10.64 ± 10.43	9.12 ± 9.08	0.290	0.157 (small)	−1.31	4.36

BMI: Body Mass Index, SR: Sit and reach, SLJ: Standing long jump, VJ: Vertical jump, HBA: Hanging with bent arm.

**Table 2:** Matrix of Pearson correlation coefficient of 2D:4D Ratio and Performance Parameters of Boy Participants.

Parameters	Digit Ratio	20 m Sprint	SR	Push Up	Sit Up	SLJ	VJ	Illinois	HBA
Digit Ratio	1								
20 m Sprint	r = −0.028 *p* = 0.784	1							
SR	r = −0.093 *p* = 0.333	r = 0.078 *p* = 0.501	1						
Push Up	r = −0.034 *p* = 0.771	r = −0.333 *p* = 0.006	r = 0.239 *p* = 0.034	1					
Sit Up	r = 0.086 *p* = 0.393	r = −0.435 *p* =< 0.001	r = 0.016 *p* = 0.875	r = 0.469 *p* =< 0.001	1				
SLJ	r = 0.191 *p* = 0.044	r = −0.725 *p* =< 0.001	r = −0.032 *p* = 0.736	r = 0.277 *p* = 0.012	r = 0.454 *p* =< 0.001	1			
VJ	r = 0.106 *p* = 0.268	r = −0.649 *p* =< 0.001	r = −0.244 *p* = 0.009	r = 0.138 *p* = 0.221	r = 0.320 *p* = 0.001	r = 0.788 *p* =< 0.001	1		
Illinois	r = −0.326 *p* = 0.029	r = 0.718 *p* =< 0.001	r = −0.205 *p* = 0.210	r = −0.362 *p* = 0.033	r = −0.216 *p* = 0.174	r = −0.553 *p =<* 0.001	r = −0.353 *p* = 0.023	1	
HBA	r = 0.304 *p* = 0.007	r = −0.483 *p* =< 0.001	r = 0.081 *p* = 0.479	r = 0.565 *p* =< 0.001	r = 0.385 *p* = 0.001	r = 0.408 *p* =< 0.001	r = 0.387 *p* = 0.001	r = −0.529 *p* = 0.001	1

SR: Sit and reach, SLJ: Standing long jump, VJ: Vertical jump, HBA: Hanging with bent arm.

**Table 3:** Matrix of Pearson correlation coefficient of 2D:4D Ratio and Performance Parameters in Female Participants.

Parameters	Digit Ratio	20 m Sprint	SR	Push Up	Sit Up	SLJ	VJ	Illinois	HBA
Digit Ratio	1								
20m Sprint	r = 0.053 *p* = 0.572	1							
SR	r = −0.040 *p* = 0.648	r = −0.278 *p* = 0.007	1						
Push Up	r = −0.072 *p* = 0.521	r = −0.281 *p* = 0.020	r = 0.163 *p* = 0.133	1					
Sit Up	r = 0.080 *p* = 0.375	r = −0.504 *p* =< 0.001	r = 0.207 *p* = 0.017	r = 0.438 *p* =< 0.001	1				
SLJ	r = 0.115 *p* = 0.192	r = −0.665 *p* =< 0.001	r = 0.271 *p* = 0.001	r = 0.259 *p* = 0.017	r = 0.604 *p* =< 0.001	1			
VJ	r = 0.047 *p* = 0.592	r = −0.651 *p* =< 0.001	r = 0.211 *p* = 0.013	r = 0.129 *p* = 0.235	r = 0.437 *p* =< 0.001	r = 0.710 *p* =< 0.001	1		
Illinois	r = 0.189 *p* = 0.219	r = 0.699 *p* =< 0.001	r = −0.401 *p* = 0.007	r = −0.358 *p* = 0.044	r = −0.405 *p* = 0.006	r = −0.585 *p* =< 0.001	r = −0.542 *p* =< 0.001	1	
HBA	r = 0.077 *p* = 0.463	r = −0.296 *p* = 0.013	r = 0.340 *p* = 0.001	r = 0.675 *p* =< 0.001	r = 0.445 *p* =< 0.001	r = 0.371 *p* =< 0.001	r = 0.297 *p* = 0.003	r = −0.330 *p* = 0.086	1

SR: Sit and reach, SLJ: Standing long jump, VJ: Vertical jump, HBA: Hanging with bent arm.

## Data Availability

The datasets generated and analyzed during the current study are not publicly available because they contain identifiable information about child participants that cannot be fully anonymized in compliance with the ethical approval granted by the Bandırma Onyedi Eylül University Health Sciences Ethics Committee (approval no. 2023/24). However, de-identified data supporting the findings of this study can be obtained from the corresponding author upon reasonable request and after confirmation that data use complies with institutional and ethical regulations.
